# Supplementary material for: A Salvaging Strategy Enables Stable Metabolite Provisioning among Free-Living Bacteria
Source: mSystems. 2022 Aug 4;7(4):e00288-22. doi: 10.1128/msystems.00288-22 (PMC9426567; doi:10.1128/msystems.00288-22)
Supplement: TABLE S1 [file msystems.00288-22-st001.docx]

| Phylum | Producers | Salvagers | Dependents | Independents |
| --- | --- | --- | --- | --- |
|  |  |  |  |  |
| Nitrospinae | 0 | 2 | 8 | 0 |
| Candidatus Azambacteria | 0 | 0 | 10 | 0 |
| Candidatus Curtissbacteria | 0 | 0 | 9 | 1 |
| Candidatus Saccharibacteria | 0 | 0 | 2 | 8 |
| Chlorobi | 0 | 10 | 0 | 0 |
| Candidatus Sungbacteria | 0 | 0 | 6 | 5 |
| Ignavibacteriae | 0 | 0 | 12 | 0 |
| Candidatus Magasanikbacteria | 0 | 0 | 8 | 4 |
| Candidatus Staskawiczbacteria | 0 | 0 | 4 | 9 |
| Candidatus Taylorbacteria | 0 | 0 | 6 | 8 |
| Candidatus Gottesmanbacteria | 0 | 0 | 13 | 2 |
| Candidatus Omnitrophica | 0 | 2 | 14 | 0 |
| Candidatus Doudnabacteria | 0 | 0 | 15 | 1 |
| Candidatus Zambryskibacteria | 0 | 0 | 17 | 0 |
| Elusimicrobia | 1 | 3 | 12 | 1 |
| Candidatus Kaiserbacteria | 0 | 0 | 2 | 15 |
| Candidatus Uhrbacteria | 0 | 0 | 16 | 2 |
| Fibrobacteres | 0 | 1 | 18 | 0 |
| Candidatus Peregrinibacteria | 0 | 0 | 18 | 1 |
| Candidatus Daviesbacteria | 0 | 0 | 16 | 4 |
| Synergistetes | 6 | 9 | 5 | 0 |
| Aquificae | 0 | 0 | 13 | 8 |
| Candidatus Levybacteria | 0 | 0 | 17 | 5 |
| Nitrospirae | 8 | 7 | 6 | 1 |
| Candidatus Woesebacteria | 0 | 0 | 19 | 4 |
| Candidatus Giovannonibacteria | 0 | 0 | 19 | 8 |
| Candidatus Roizmanbacteria | 0 | 0 | 21 | 7 |
| Verrucomicrobia | 0 | 2 | 26 | 1 |
| Candidatus Yanofskybacteria | 0 | 0 | 34 | 0 |
| Thermotogae | 2 | 9 | 24 | 0 |
| Planctomycetes | 8 | 1 | 28 | 0 |
| Acidobacteria | 4 | 7 | 28 | 0 |
| Chlamydiae | 0 | 0 | 16 | 26 |
| Chloroflexi | 11 | 16 | 19 | 0 |
| Fusobacteria | 44 | 4 | 0 | 2 |
| Deinococcus-Thermus | 15 | 23 | 14 | 0 |
| Spirochaetes | 17 | 11 | 40 | 30 |
| Tenericutes | 0 | 1 | 26 | 115 |
| Cyanobacteria | 203 | 2 | 20 | 2 |
| Bacteroidetes | 6 | 324 | 689 | 21 |
| Actinobacteria | 1022 | 38 | 552 | 195 |
| Firmicutes | 646 | 473 | 744 | 275 |
| Proteobacteria | 2180 | 974 | 1423 | 258 |
