# Supplementary figures and images for: A Salvaging Strategy Enables Stable Metabolite Provisioning among Free-Living Bacteria
Source: mSystems. 2022 Aug 4;7(4):e00288-22. doi: 10.1128/msystems.00288-22 (PMC9426567; doi:10.1128/msystems.00288-22)

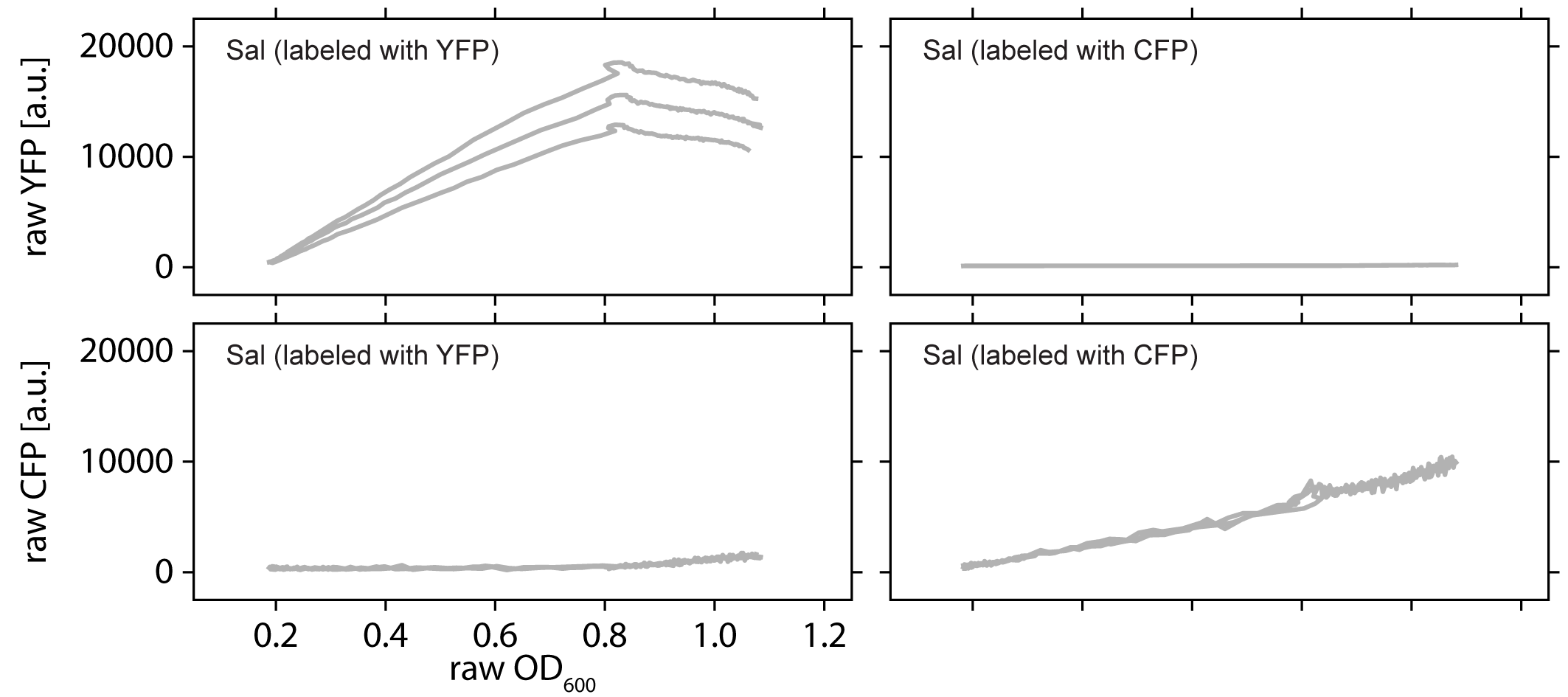

Supplement: FIG S1 [file msystems.00288-22-sf001.tif]

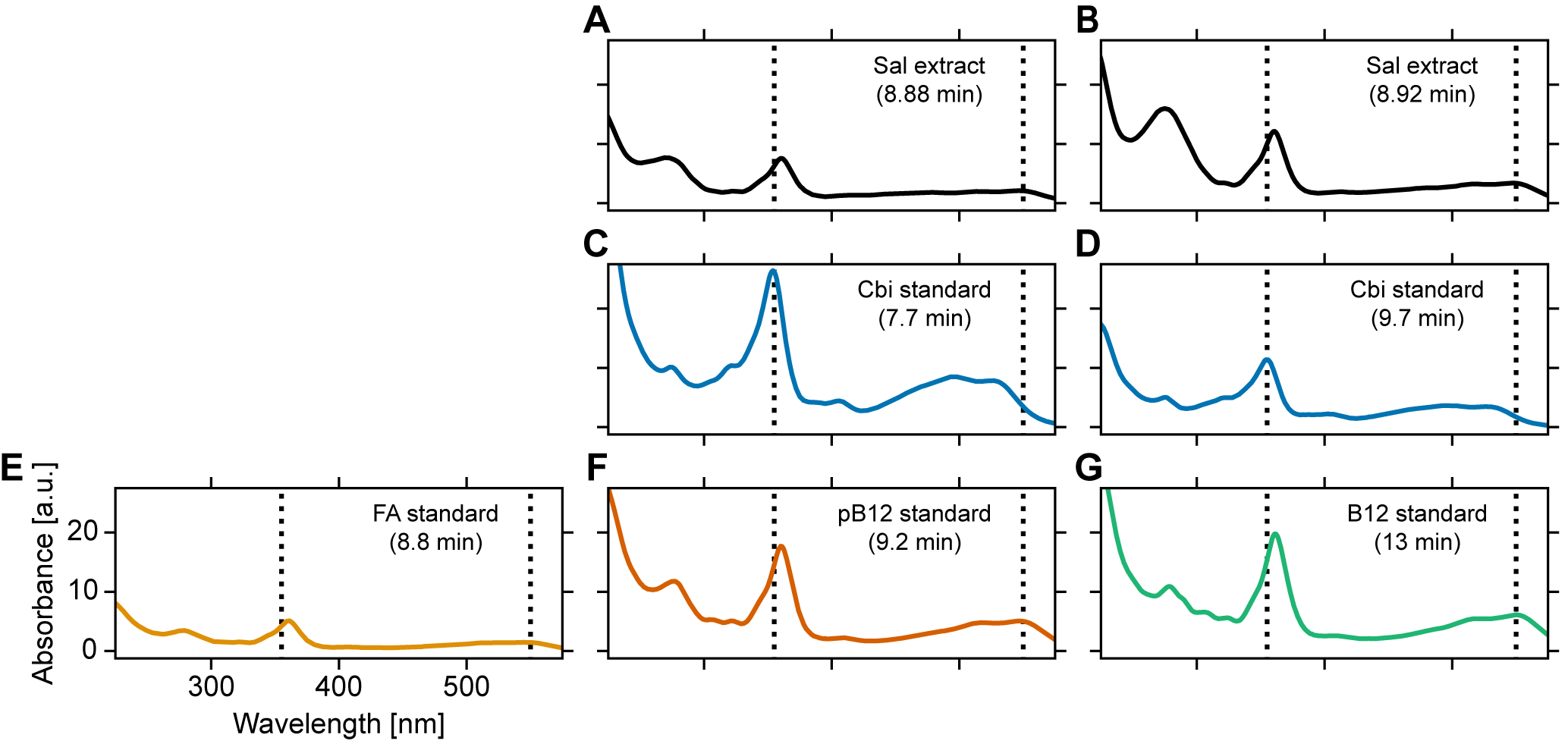

Supplement: FIG S4 [file msystems.00288-22-sf004.tif]

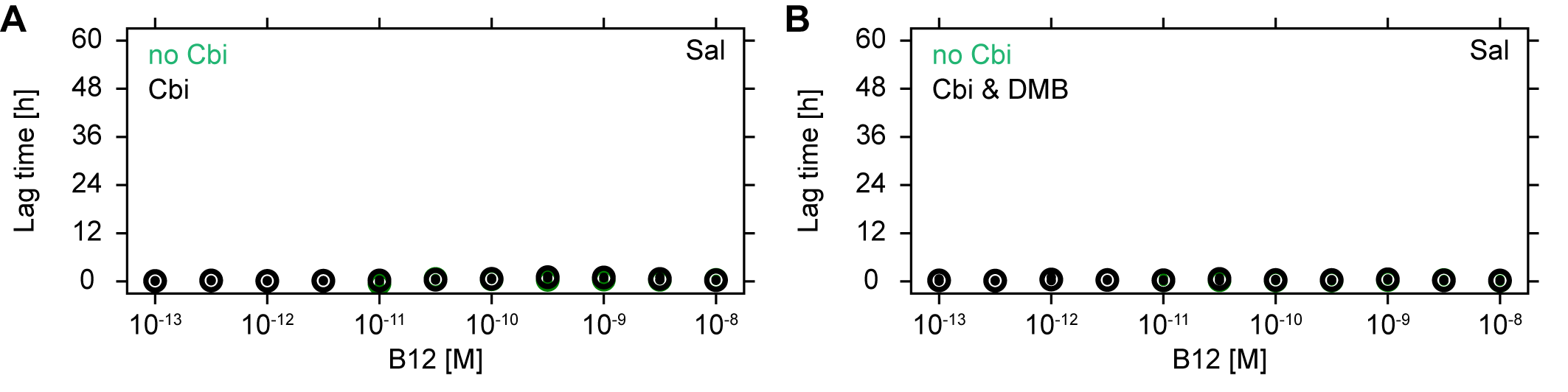

Supplement: FIG S2 [file msystems.00288-22-sf002.tif]

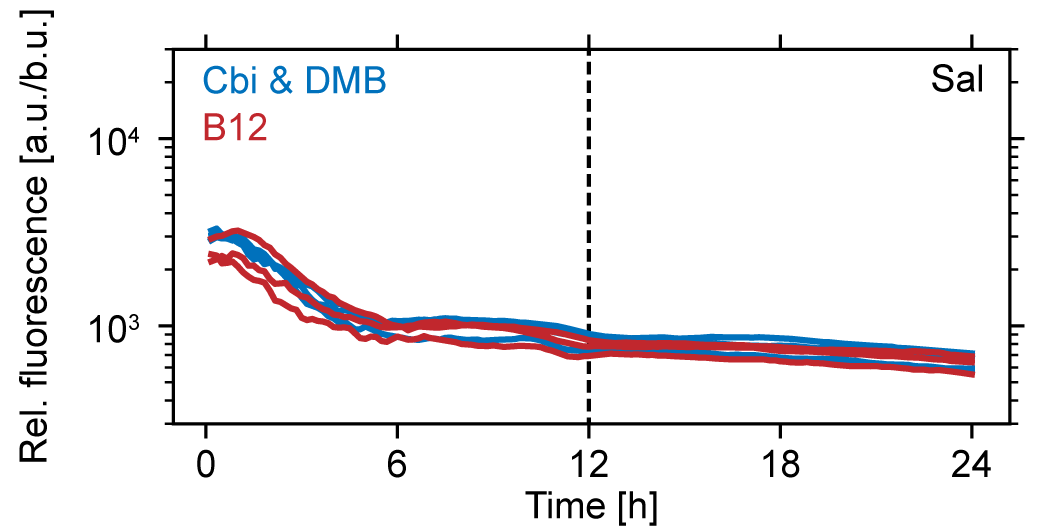

Supplement: FIG S3 [file msystems.00288-22-sf003.tif]

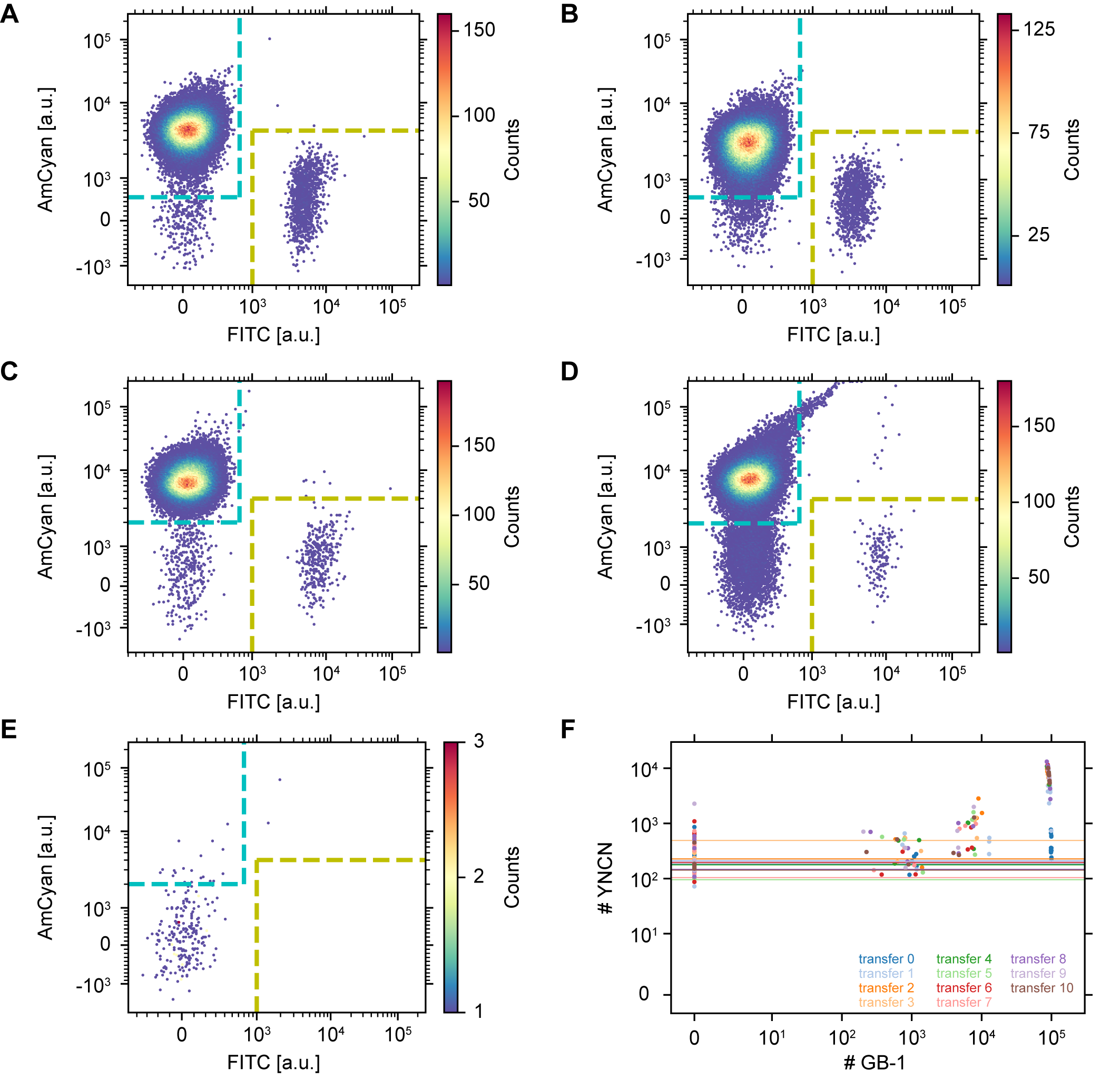

Supplement: FIG S5 [file msystems.00288-22-sf005.tif]
