# Supplementary material for: A Salvaging Strategy Enables Stable Metabolite Provisioning among Free-Living Bacteria
Source: mSystems. 2022 Aug 4;7(4):e00288-22. doi: 10.1128/msystems.00288-22 (PMC9426567; doi:10.1128/msystems.00288-22)
Supplement: TABLE S2 [file msystems.00288-22-st002.docx]

| **name** | **genotype** | **source** |
| --- | --- | --- |
|  |  |  |
| Sal+ | *Escherichia coli* MG1655 ΔmetE | <https://doi.org/10.1128/AEM.02117-19> |
| Sal- | *Escherichia coli* MG1655 ΔmetE ΔcobUST ΔcobC | <https://doi.org/10.1128/mBio.02507-20> |
| Sal+^GB-1^ | *Escherichia coli* MG1655 GB-1 ΔmetE | This study. |
| Sal-^GB-1^ | *Escherichia coli* MG1655 GB-1 ΔmetE ΔcobUST ΔcobC | This study. |
