# Supplementary material for: A Salvaging Strategy Enables Stable Metabolite Provisioning among Free-Living Bacteria
Source: mSystems. 2022 Aug 4;7(4):e00288-22. doi: 10.1128/msystems.00288-22 (PMC9426567; doi:10.1128/msystems.00288-22)
Supplement: TABLE S3 [file msystems.00288-22-st003.docx]

| **name** | **backbone** | **insert** | **source** |
| --- | --- | --- | --- |
|  |  |  |  |
| pSG013 | Kan^R^ marker, pBR322 origin, and rop gene | constitutive promoter (BBa_J23100, iGEM), RBS (BBa_B0034, iGEM), and mCerulean (QB3 MacroLab, UC Berkeley) | This study. |
| pSG015 | Kan^R^ marker, pBR322 origin, and rop gene | constitutive promoter (BBa_J23100, iGEM), RBS (BBa_B0034, iGEM), and mCitrine (QB3 MacroLab, UC Berkeley) | This study. |
| pSG033 | Kan^R^ marker, pBR322 origin, and rop gene | cobUST regulatory region (genomic region 500 bp upstream of cobUST start codon in *E. coli* MG1655 wt) and mCitrine (QB3 MacroLab, UC Berkeley) | This study. |
